# The Expression and Function of CD300 Molecules in the Main Players of Allergic Responses: Mast Cells, Basophils and Eosinophils

**DOI:** 10.3390/ijms21093173

**Published:** 2020-04-30

**Authors:** Joana Vitallé, Iñigo Terrén, Ane Orrantia, Agurtzane Bilbao, Pedro M. Gamboa, Francisco Borrego, Olatz Zenarruzabeitia

**Affiliations:** 1 Immunopathology Group, Biocruces Bizkaia Health Research Institute, 48903 Barakaldo, Spain; joanagorliz@hotmail.com (J.V.); inigo.terrenmartinez@osakidetza.eus (I.T.); ane.orrantiarobles@osakidetza.eus (A.O.); agurtzane.bilbaoaburto@osakidetza.eus (A.B.); pedromanuel.gamboasetien@osakidetza.eus (P.M.G.); francisco.borregorabasco@osakidetza.eus (F.B.); 2 Pediatrics Service, Cruces University Hospital, 48903 Barakaldo, Spain; 3 Allergology Service, Cruces University Hospital, 48903 Barakaldo, Spain; 4 Ikerbasque, Basque Foundation for Science, 48013 Bilbao, Spain

**Keywords:** allergy, CD300 receptors, mast cells, basophils, eosinophils, atopic dermatitis, IgE, FcɛRI, phosphatidylserine, phosphatidylethanolamine, ceramide

## Abstract

Allergy is the host immune response against non-infectious substances called allergens. The prevalence of allergic diseases is increasing worldwide. However, while some drugs counteract the symptomatology caused by allergic reactions, no completely effective treatments for allergic diseases have been developed yet. In this sense, the ability of surface activating and inhibitory receptors to modulate the function of the main effector cells of allergic responses makes these molecules potential pharmacological targets. The CD300 receptor family consists of members with activating and inhibitory capabilities mainly expressed on the surface of immune cells. Multiple studies in the last few years have highlighted the importance of CD300 molecules in several pathological conditions. This review summarizes the literature on CD300 receptor expression, regulation and function in mast cells, basophils and eosinophils, the main players of allergic responses. Moreover, we review the involvement of CD300 receptors in the pathogenesis of certain allergic diseases, as well as their prospective use as therapeutic targets for the treatment of IgE-dependent allergic responses.

## 1. Introduction

Allergic diseases, which are defined as anomalous adaptive immune responses to non-infectious substances named allergens, or in other words, immunologically-mediated and allergen-specific hypersensitivities, are on the rise for more than 50 years, especially in the industrialized world [1,2]. According to the American Academy of Allergy, Asthma and Immunology (AAAAI), between 20% and 30% of people worldwide are affected by allergies, while among school children, sensitization rates to one or more common allergens are currently approaching 40%–50%. Allergic processes begin with the sensitization to an allergen and conclude with allergic inflammation, which consist of an early-phase reaction, followed by a late-phase reaction in many subjects. When the exposure to the allergen persists, a chronic allergic inflammation could be developed, which may result in tissue alterations and remodeling. Several immune cell types take part in these different stages; in particular, T and B lymphocytes, mast cells, basophils and eosinophils have a crucial role in allergic processes, which are in part regulated by a vast array of activating and inhibitory cell surface receptors [3,4,5,6,7,8,9,10].

## 2. IgE-Mediated Allergic Responses

In IgE-mediated allergic processes, the individual is firstly sensitized to an allergen. In this phase, antigens are captured and presented by antigen-presenting cells to naïve T cells, inducing their differentiation to T helper type 2 (Th2) cells. These Th2 cells engage cognate B cells and secrete interleukin (IL)-4 and IL-13, which causes the class-switch recombination of B cells, resulting in an antigen-specific IgE production [3,7,11,12]. Afterwards, IgE binds, among others, to FcεRI, the high-affinity surface receptor for IgE, expressed on the surface of mast cells and basophils. These myeloid cells share common features, such as their key role in infections and autoimmune and allergic disorders. However, they differ in their maturation and location. Mast cells derive from progenitor cells which leave the bone marrow and mature within peripheral tissues, while basophils completely mature in the bone marrow and circulate in the blood [7,12,13]. The involvement of both mast cells and basophils in IgE-mediated allergies is directly related to the expression of FcεRI on their cell surface, which is bound to IgE after the sensitization process and leads to allergic inflammation.

Once the individual is sensitized, the re-exposure to the same bivalent or multivalent antigen can cause early-phase reactions within a few minutes, which could be localized or systemic (anaphylaxis). In these reactions, the allergen binds to IgE and cross-links the FcεRI on mast cells and basophils, inducing the activation of a complex signaling cascade that results in the release of a wide spectrum of biologically active products such as histamine and serotonin, lipid-derived mediators (e.g., prostaglandin D2 and cysteinyl leukotrienes (LT)) and various cytokines, chemokines and growth factors (e.g., tumor necrosis factor (TNF) and vascular endothelial growth factor A (VEGFA)). This phase is clinically characterized by vasodilation, increased vascular permeability and alterations in affected organs [3,13,14,15,16]. The secretion of the different substances during the early-phase reaction leads to the recruitment and activation of inflammatory cells and particularly Th2 cells, eosinophils and basophils, resulting in a persistent production of mediators mainly by mast cells or T cells. This persistent mediator production promotes the late-phase reaction, which causes, for instance, airway narrowing and mucus hyper-secretion in the lungs. When the specific allergen is exposed in a prolonged or repetitive manner, a chronic allergic inflammation could be originated [5,6,12,16]. This phase can go on for months or years and is characterized, not only by the presence of high numbers of leukocytes (mainly eosinophils and Th2 cells), but also is usually associated with structural alterations to the affected tissues. In tissue sites of allergic inflammation, a cross-talk between eosinophils and mast cells takes place, defined as an allergic effector unit, which promotes the amplification and persistence of allergic reactions [17,18].

Eosinophils are myeloid cells that, once they are matured in the bone marrow under the influence of IL-3, IL-5 and granulocyte macrophage–colony stimulating factor (GM-CSF), move to the peripheral blood and circulate there until they are recruited to inflammation sites by different stimuli. These cells are characterized by their cytoplasmic granules containing cationic proteins that function as growth factors and inflammatory mediators, such as the major basic protein (MBP) and eosinophil-derived neurotoxin (EDN), and produce the cytokine stem cell factor (SCF) and nerve growth factor (NGF), among others, which induce mast cell survival and maturation [19]. The mechanism of action not only of eosinophils, but also of mast cells and basophils during the different phases of allergic inflammation is regulated by a variety of immune receptors transmitting stimulatory and inhibitory signals, such as CD300 family receptors, which are being widely studied in order to find effective therapeutic targets [7,8,9].

## 3. CD300 Receptor Family

The CD300 molecules constitute an evolutionarily significant family of receptors mainly expressed on the surface of human and mouse immune cells [20,21,22,23,24,25,26,27]. In humans, the CD300 family consists of eight members encoded by the genes *CD300A, B, C, D, E, F, G*, and *H* and they are divided into two groups depending on their activating or inhibitory function. All of them are type I transmembrane proteins formed by an immunoglobulin (Ig)V-like extracellular domain and a cytoplasmic tail, which could be short or long depending on their signaling capacity. The majority of these receptors (CD300b, CD300c, CD300d, CD300e and CD300h) have a short cytoplasmic tail without functional signaling domains, and instead, they have a charged transmembrane residue that allows the association with adaptor proteins containing immunoreceptor tyrosine-based activating motifs (ITAMs) such as DNAX-activating protein (DAP)12 and Fc receptor (FcR)γ chain, or phosphatidylinositol 3-kinases (PI3K) binding motifs (YxxM) such as DAP10, providing them a stimulatory or co-stimulatory function. Ligand binding to the activating receptors results in the phosphorylation of tyrosine-based motifs present in the associated adaptor molecules, which is required for further recruitment of protein-tyrosine kinases such as Syk, ZAP-70 or PI3K that will stimulate a series of intracellular events inducing cell differentiation, growth and survival, adhesion, migration, phagocytosis, cytokine production and/or cytotoxicity [28]. By contrast, CD300a and CD300f contain a long cytoplasmic tail with immunoreceptor tyrosine-based inhibitory motifs (ITIMs), displaying an inhibitory capacity [20,21,23,25,26,27,29]. Tyrosine phosphorylation of the ITIMs is required for the transmission of the inhibitory signal. Then, phosphorylated ITIMs will recruit different phosphatases depending on the cell type. For example, whereas in mouse bone marrow-derived mast cells (BMMCs), both Src homology 2 domains containing protein tyrosine phosphatase (SHP)-1 and SHP-2 are recruited to the phosphorylated ITIMs of CD300f inducing an inhibitory signal [30], a dominant role for SHP-1 has been suggested in human CD300a- and CD300f-mediated inhibitory signals [31,32,33]. In the case of CD300f, although it has been classically considered as an inhibitory receptor, it has been demonstrated that it is also able to transmit activating signals through PI3K-binding motifs and growth factor receptor-bound protein 2 (Grb2) [33,34]. Although the members of the CD300 family mentioned until now display the previously described structure, the exception is the CD300g receptor, which instead of having inhibitory or activating motifs, has, in addition to the IgV-like domain, an extracellular mucin-like domain and is expressed in endothelial cells [35]. In mice, the CD300 family includes nine members which are encoded by nine genes located on chromosome 11, the synthenic region of human chromosome 17 [21,23,26]. As in humans, mouse CD300f possesses ITIM motifs as well as Grb2 and PI3K-binding domains in its cytoplasmic tail [30,36,37,38]. Furthermore, mouse CD300f has also been demonstrated to associate with the ITAM-containing adaptor FcRγ chain [30].

Although further research is required in order to discover the specific ligands of each CD300 family member, it is already known that several CD300 receptors, such as CD300a, CD300c and CD300f, recognize the aminophospholipids phosphatidylserine (PS) and phosphatidylethanolamine (PE), which are exposed in the outer leaflet of the plasma membrane of activated, infected, transformed or apoptotic cells [39,40,41,42,43,44,45,46]. Both CD300a and CD300c receptors recognize PS and PE, although the affinity of each one is different. CD300c recognizes both phospholipids with a similar affinity and its binding to PS is also similar to the one of CD300a [42,44]; however, human CD300a binds PE with higher affinity than PS [41]. Other CD300 receptors such as CD300b and CD300f are also able to bind PS [39,43], although they also recognize other ligands. For example, CD300b binds lipopolysaccharide (LPS) [47]. Regarding CD300f, it has also been shown that it recognizes ceramide and sphingomyelin [48,49,50]. Moreover, CD300e has been demonstrated to recognize sphingomyelin [51].

Over the last few years, the biological and clinical significance of CD300 molecules and their participation in the pathogenesis of numerous diseases such as allergy, psoriasis, colitis, multiple sclerosis, leukemia, sepsis, infection diseases, etc. have been well documented [21,23,25,52,53,54,55,56,57,58,59,60,61]. In this review, our main objective is to describe the current knowledge of the expression and function of CD300 molecules in key effector cells of allergic reactions, specifically mast cells, basophils and eosinophils (Table 1), which have an essential role in the effector phases of allergic responses. Understanding the role of CD300 molecules in the modulation of allergic diseases would help to develop new anti-allergy therapies.

## 4. CD300 Receptor Family in Mast Cells, Basophils and Eosinophils

### 4.1. CD300a

CD300a, one of the inhibitory members of the CD300 receptor family, is constitutively expressed in mast cells [62], basophils [57] and eosinophils [63], the three main effector cell types in allergic reactions. The expression levels vary in response to several stimuli. Thereby, CD300a expression on human basophils is rapidly up-regulated in response to IgE/FcεRI cross-linking [57,64]. In addition, stimulation with N-formylmethionyl-leucyl-phenylalanine (fMLP), phorbol 12-myristate 13-acetate (PMA) or PMA plus ionomycin also induce a significant up-regulation of CD300a, but at lower levels compared to the one induced via IgE/FcεRI [57]. Incubation of basophils with IL-3, IL-10 and transforming growth factor beta-1 (TGF-β1) neither affects the expression of CD300a on basophils nor modifies allergen-induced CD300a up-regulation [57]. Regulation of CD300a expression has also been demonstrated in mast cells, where it is known that eosinophil-derived MBP and EDN down-regulate CD300a expression on cord blood-derived mast cells (CBMCs) [62]. In the case of eosinophils, the expression of CD300a is up-regulated by hypoxia and GM-CSF [65], and the inhibition of hypoxia inducible factor (HIF)-1 abolishes the hypoxia/GM-CSF-induced CD300a increase [65]. In a mouse model of allergic peritonitis, an allergen-driven selective increase in CD300a has been described in eosinophils at 18 h, peaking at 3–4 days, after allergen challenge initiation [66]. In eosinophils, while the overnight increase in CD300a described by Karra et al. would imply de novo synthesis, probably through extracellular signal-regulated kinases (ERK)1/2 phosphorylation, the short-term (by 3 h) increase described in response to GM-CSF would rather involve a rapid mechanism of receptor transport to the membrane [64]. Something similar has been described in human basophils in response to IgE/FcεRI cross-linking where the expression of CD300a is rapidly up-regulated (within minutes) [57,64]. The notion that an intracellular pool of CD300a might be maintained for fast translocation after cell stimulation [67] suggests the importance of this receptor for the prompt regulation of cellular functions.

As regards to the function of CD300a on mast cells, in vitro analyses have demonstrated that cross-linking of CD300a with monoclonal antibodies (mAb) suppresses FcεRI-mediated signals, resulting in a decreased degranulation in human and mouse mast cells [68,69]. However, CD300a did not influence the IgE-independent activation of human CBMC [62]. Cross-linking of CD300a on the surface of human eosinophils significantly inhibited their chemotaxis, survival, and activation in response to eotaxin, GM-CSF and IL-5 [63]. In addition, CD300a is able to inhibit LTB_4_-induced eosinophil transmigration. However, cross-linking of CD300a does not inhibit TNF-mediated activation of eosinophils. It is possible that CD300a cross-linking may block Src kinase-dependent activation pathways, leaving others signaling pathways, such as TNF receptor family, unaffected [63]. It has also been demonstrated that the apoptosis-related PS exposure suppresses human basophil anaphylactic degranulation via the inhibitory receptor CD300a. In fact, cross-linking of CD300a with PS results in a significant dose-dependent inhibition of IgE/FcεRI-mediated cell activation [64]. Moreover, at least in mast cells, a *cis*-interaction, rather than a *trans*-interaction, of CD300a with PS has been described [70]. This *cis*-interaction regulates FcεRI-mediated mast cell degranulation in vivo as well as in vitro, adding another layer of regulation in allergic responses [70]. Recently, the important role of CD300a in allergic inflammation and its resolution was described in a CD300a−/− mouse model of allergic peritonitis. CD300a-deficient mice displayed a rapid augmentation of inflammatory cell infiltrates and tryptase content in the peritoneal cavity, and the resolution process was delayed, in comparison with wild type (WT) mice [66]. Briefly, CD300a has been shown to downregulate mast cells, eosinophils and basophils effector functions, thereby serving as a potential target for inhibiting allergic effector cells input in allergy. In addition, because many cells undergo apoptosis at the site of allergic inflammation [71,72,73], and considering the PS-CD300a-based self-regulation capacity of, at least, mast cells, CD300a may play an important anti-inflammatory role in allergic responses and may be a potential prophylactic and/or therapeutic target for the treatment of allergic diseases.

### 4.2. CD300f

The expression of CD300f has also been demonstrated in mast cells [59,74], eosinophils [75,76] and basophils [58]. Interestingly, allergen challenge causes a significant increase in CD300f expression in alveolar macrophages, eosinophils and mast cells in vivo [59]. The regulation of the expression of CD300f in basophils has been also studied in response to several IgE-dependent and IgE-independent basophil activators, including anti-IgE Ab, IL-3, thymic stromal lymphopoietin (TSLP), IL-33, and fMLP without observing any significant change in its expression levels [58].

In human eosinophils, CD300f has been identified as a novel and specific negative regulator of eotaxin-induced responses including migration, calcium influx, actin polymerization and intracellular signaling [75]. CD300f, through its binding to PS exposed by apoptotic eosinophils, is capable of suppressing eosinophils chemotaxis, at least in vitro [75], suggesting that recruitment of eosinophils to sites of tissue damage and cell death is actively regulated by intrinsic negative feedback mechanisms such as CD300f. Besides its classical role as an inhibitory receptor, the dual functions, inhibitory and activating, of CD300f have been demonstrated, not only in eosinophils, but also in mast cells [74]. On the one hand, the binding of CD300f to its physiological ligands ceramide and sphingomyelin inhibits IgE-mediated mast cell activation and allergic responses, including passive cutaneous anaphylaxis (PCA) responses [48,49]. Moreover, it has been shown that CD300f deficiency in mice exacerbates mast cell-dependent allergic responses, including anaphylaxis, airway inflammation and dermatitis, and food allergic responses [48,77,78,79]. Recently, using ovalbumin (OVA)-induced IgE- and mast cell-dependent food allergy mouse models, it was demonstrated that the interaction between CD300f and ceramide inhibits food allergic responses presumably by suppressing IgE-mediated activation of mast cells [79]. The inhibitory role of CD300f has been also described on human mast cells, where it was found that the interaction between extracellular ceramide and CD300f suppresses human mast cell-dependent allergic responses [49]. In fact, the extracellular lipid ceramide has been suggested as an anti-allergic tissue component able to inhibit mast cell-dependent allergic responses through its binding to CD300f [48,49]. On the other hand, CD300f has been identified as an IL-4-induced molecule [59], and interestingly, it was demonstrated that CD300f is co-localized and physically associated with IL-4Rα both under baseline conditions and following IL-4 stimulation [59]. It has been described that CD300f amplifies IL-4Rα-induced responses, and indeed impaired IL-4-induced activation has been observed in CD300f−/− mast cells, eosinophils and dendritic cells, demonstrating that CD300f amplifies the IL-4/IL-13-induced signaling, mediator release and priming [59]. In fact, IL-4- and aeroallergen-treated mice lacking CD300f exhibited less IgE production, chemokine expression and inflammatory cell recruitment than WT mice [59].

### 4.3. Other CD300 Molecules

Although several groups are studying the relevance of the inhibitory receptors CD300a and CD300f in the regulation of allergic processes, less is known about the expression, regulation and function of other CD300 molecules such as CD300b, CD300c and CD300d in mast cells, eosinophils and basophils.

The activating receptor CD300b has been identified on the surface of mouse and human mast cells [80]; however, very little is known about its role in allergic reactions. It was demonstrated to have an activating role in BMMCs, as the cross-linking of transduced mouse CD300b caused activation events, including cytokine production, cell survival, degranulation, and adhesion to the extracellular matrix [80]. Mouse CD300b associates with the adaptor protein DAP12, and to a lesser extent with DAP10, and it has been shown that CD300b-mediated functions of BMMCs are strongly dependent on DAP12. Unlike mouse CD300b, cross-linking of human CD300b expressed in mouse BMMCs induces cytokine production even in the absence of both DAP12 and DAP10, suggesting the existence of unidentified adaptors that initiate the activating signaling cascade in humans. Interestingly, as opposed to mouse CD300b, human CD300b possesses a tyrosine residue (Y188) in the cytoplasmic region, which is a docking site for the intracellular signaling mediator Grb2 [81]. When human CD300b is ectopically expressed in mouse cells, signaling via Y188 phosphorylation plays a predominant role in cytokine production in DAP12-deficient, but not WT mast cells. In addition, experiments using DAP10/DAP12 double-deficient mouse BMMCs suggest the existence of Y188 phosphorylation-dependent and -independent signals from unidentified adaptors [80]. Collectively, although the CD300b-mediated effector functions seem to be differentially regulated in mouse versus human mast cells, it plays an activating role in both of them. Unexpectedly, despite having similar surface expression levels of CD300b, cross-linking of endogenous mouse CD300b induces a more pronounced activation in fetal liver mast cells than in BMMCs [80], suggesting that CD300b plays an important role in specific types of mast cells, closely related to their differentiation and tissue distribution.

In the case of CD300c, its constitutive expression has been demonstrated in human mast cells [42] and basophils [53,58], although little is known about the regulation of this expression. The only published data have demonstrated that the expression of CD300c on basophils is regulated in response to IL-3, a cytokine strongly linked to the IgE-dependent basophil activation [82]. It has been shown that IL-3 induces a significant increase in the expression of CD300c after 18 h of stimulation but not in a short-term stimulation, suggesting that IL-3-mediated upregulation of CD300c expression involves transcriptional and/or translational mechanisms [58]. Regarding functional properties, the Ab-mediated cross-linking of CD300c on human mast cells induced the production of significant levels of IL-8 protein and CCL1 mRNA, implicating CD300c as an activating receptor in these cells [42]. In addition, it has been demonstrated that CD300c acts as a co-stimulatory molecule during basophil activation, increasing IgE-mediated basophil degranulation and cytokine production [58].

Apart from CD300a, CD300b, CD300c, and CD300f, mouse mast cells also express CD300d and CD300h. CD300d has been demonstrated to activate them in a Lyn- and Syk-dependent manner, resulting in the secretion of newly synthesized and preformed chemical mediators [74]. It has been demonstrated that CD300d ligation strongly enhances LPS-induced cytokine production of mouse mast cells and granulocytes. In addition, LPS stimulation leads to a down-regulation of CD300d expression [74]. In the case of CD300h, which shares a high homology with CD300d, it has been shown to transmit an activating signal through interaction with FcRγ, inducing a cytokine production in BMMCs [83].

## 5. CD300 Molecules in Allergic Individuals

**Figure 1:**
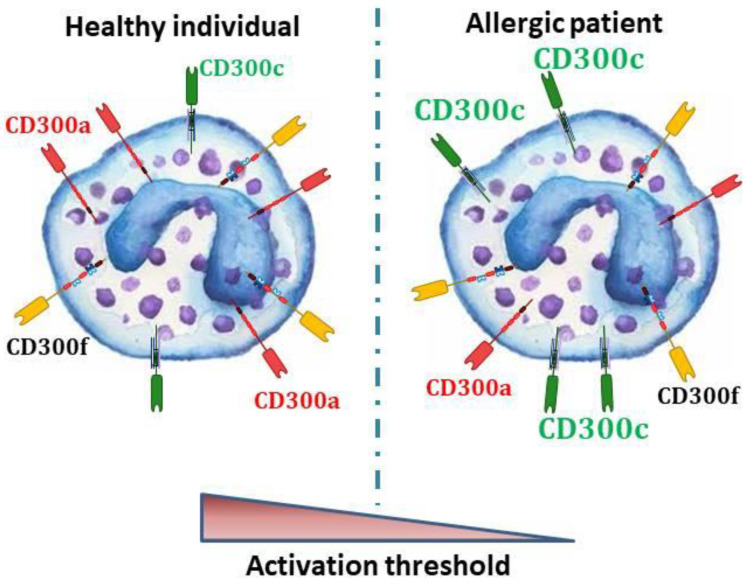
In human basophils, IgE-dependent activation is regulated, among others, by CD300 activating and inhibitory receptors. In allergic individuals, a lower expression of the CD300a inhibitory receptor [57] and a higher expression of CD300c activating receptor [53,58] have been described. Therefore, it could be postulated that in allergic people, the unbalance in the CD300 mediated signals may lead to a lower FcεRI activation threshold.

In spite of knowing that several CD300 receptors are expressed on the surface of the key players in allergic reactions, little is known about the significance of this expression in the pathophysiology of allergic diseases in humans. The few studies in allergic patients have suggested a very interesting role of CD300 molecules in the modulation of the activation threshold of basophils (Figure 1), eosinophils, and probably mast cells, during allergic reactions.

On the one hand, a decreased basal expression of the CD300a inhibitory receptor on basophils from birch pollen-allergic patients in comparison with the ones from healthy donors has been described [57]. Remarkably, it was demonstrated that CD300a expressed on basophils suppresses the basophil anaphylactic degranulation by its interaction with PS and PE exposed on apoptotic cells [58,64]. Differences in CD300c expression levels have also been described between allergic and non-allergic individuals. Thus, an overexpression of CD300c was observed on basophils from cow’s milk-allergic children [58] and also on basophils from dust mites- and grass pollen-allergic individuals [53], three types of IgE-dependent allergies. In addition, the potential use of CD300c as a biomarker for the diagnosis and stratification of allergic patients was recently suggested since its expression intensity has been associated to the severity of the hypersensitivity symptoms in allergic children [58]. On the other hand, peripheral blood eosinophils in allergic rhinitis patients revealed significantly elevated levels of CD300f in comparison with healthy individuals [59]. Despite the differences observed in basophils and eosinophils, in a recent study carried out with human peripheral blood-derived mast cells (PBdMC) generated from circulating CD34+ hematopoietic stem cells, no differences were observed in the molecular, including CD300a expression, and stimulus-response profiles of PBdMC from peanut allergic and non-allergic subjects [84].

As far as regulation is concerned, after cross-linking with either anti-IgE Ab or specific allergens, basophils from allergic patients exhibit a significant and rapid up-regulation of CD300a expression in all activated basophils that persisted for over 2 h [57]. In healthy individuals, up-regulation of CD300a occurs with similar kinetics as in patients when stimulated with anti-IgE Ab [57]. In both, patients and healthy controls, preincubation with anti-CD300a monoclonal antibody (mAb) significantly inhibit IgE-mediated CD63 expression, a widely used basophil activation marker which is rapidly mobilized to the cell surface by polyclonal anti-IgE and allergens. In contrast, IgE-independent basophil activation with fMLP, PMA, and ionomycin is not inhibited by CD300a engagement [57].

Among allergic diseases, atopic dermatitis (AD) is a predominantly Th2-driven inflammatory skin disease in which mast cell accumulation and eosinophil infiltration are typically present in AD skin lesions [85,86]. In skin diseases, the importance of CD300 molecules has been previously suggested by studies showing that the CD300 gene family is located in the PSORS2 psoriasis susceptibility locus, also linked to AD [55]. In fact, a single nucleotide polymorphism that encodes for a non-synonymous mutation (R94Q) within the extracellular domain of CD300a, and that affects to the binding to PS and PE, has been associated with psoriasis susceptibility [41,55]. Recently, it was described that CD300a expression is modulated in AD patients and that could influence the inflammatory response [54]. In fact, a significant increase in CD300a total expression was observed in AD biopsies from lesional skin when compared to normal skin. Specifically, CD300a expression was significantly increased on eosinophils and macrophages and non-significantly on mast cells [54]. Of note, authors detected a similar up-regulation of CD300a by gene array analysis of both AD skin lesions and lesional psoriatic skin samples, indicating that the up-regulation of CD300a is not unique for AD. In conclusion, and considering the overall increased skin inflammation observed in AD-induced CD300a−/− mice [54], a down-regulatory role for CD300a was suggested, not only in AD but also in other allergic diseases.

On the other hand, it is known that the content of ceramide, a physiological ligand of CD300f abundant in normal epidermis, is decreased in an AD skin [87]. Although this decrease in ceramide levels in the skin is thought to be associated with the barrier dysfunction that causes AD, it has been suggested that the mast cell-dependent inflammation observed in AD could be accelerated as a result of the lack of ceramide-CD300f interaction [48]. The inhibition of AD-like skin lesions by topical application of a ceramide derivative in mice supports this possibility [88] and suggests that strategies driven to modulate signalling through CD300 receptors could be used as therapy in allergic diseases.

## 6. CD300a as Potential Therapeutic Target in Allergic Diseases

In the treatment of allergic diseases, several drugs are commonly used in the clinical practice targeting soluble mediators secreted by mast cells and basophils, such as histamine and leukotrienes (e.g., antihistamines and LTC_4_ synthesis inhibitors or LTC receptor antagonists) [89,90]. However, they have not been effective enough for numerous allergic patients [90,91,92]. For this reason, different targets are being investigated so as to improve the therapy against allergies, including activating and inhibitory cell surface receptors expressed in both mast cells and basophils [89,93,94]. The most studied activating receptor as a target for novel therapies is FcεRI and its binding with specific IgE. In this sense, the humanized mAb omalizumab, an anti-IgE mAb, is the only approved mAb in the clinic able to block the interaction of IgE with FcεRI [95,96,97,98,99,100,101]. Omalizumab treatment reduces IgE plasma levels, as well as the expression of FcεRI on mast cells and basophils [102,103,104,105,106,107]. Moreover, other effects, such as downregulation of B cells IgE class-switching, might also contribute to its efficacy [108,109,110]. Another strategy to interrupt the FcεRI-IgE interaction is to use small peptides mimicking the sequence and structure of the Fc portion of the IgE [111,112] or the extracellular region of FcεRI α-chain [113,114]. This strategy seems promising as it has shown protective effects in experimental models [113,114]. Furthermore, it has been also proposed to target other activating molecules, including CD48, an important receptor for the initiation and continuation of allergic reaction [115], which has been described to be upregulated in two murine models of allergic eosinophilic airway inflammation [116]. TSLP, a molecule that promotes the secretion of inflammatory cytokines from mast cells [117], has also been proposed as a therapeutic target in allergic diseases. Among the inhibitory receptors, FcγRIIB and Siglec-8 have shown promising preclinical results in the context of allergy treatment [118,119,120]. FcγRIIB is expressed in mast cells and basophils, while Siglec-8 is only expressed in mast cells, and both negatively regulate the IgE-mediated activation of these cell types through ITIM-mediated signaling [118,119,121]. Apart from these molecules, in the last few years, CD300 surface receptors have also been investigated as potential therapeutic targets. Bispecific Ab fragments were generated by chemical conjugation of Fab’ fragments of anti-human IgE and CD300a and anti-mouse IgE and CD300a [122]. These constructs completely abrogated IgE-induced signaling and activation in both human and murine mast cells in vitro. Moreover, the construct targeting murine IgE and CD300a was administered simultaneously with allergen challenge in murine models of PCA and experimental asthma, showing the capacity of abolishing the allergic-inflammatory response in both of them, and thus demonstrating that specific targeting of CD300a on mast cells could be an effective therapeutic approach to inhibit allergic reactions [122]. Another bispecific Ab able to recognize CD300a and CCR3, a chemokine receptor highly expressed on basophils, eosinophils and mast cells, has been shown to inhibit eosinophils and mast cell activation and mediator release in vitro [63]. This Ab has also been seen to act against tissue inflammation and remodeling in a murine model of chronic experimental asthma [63], demonstrating that a specific targeting of CD300a in CCR3-positive cells may be a potent tool for treating airway inflammation and tissue remodeling [63]. In addition, another bispecific Ab linking Kit and CD300a has been demonstrated to inhibit SCF-induced mast cells differentiation, survival, and activation in vitro, as well as the allergic reaction induced by SCF in a murine model of cutaneous anaphylaxis [123].

## 7. Conclusions

Although further research is needed in order to elucidate the expression regulation and biological functions of, for example, CD300f in basophils, as well as the implication of CD300c in mast cell-dependent reactions, there is accumulating evidence that CD300-mediated signals are able to modulate the activation of basophils, eosinophils and mast cells in allergic responses promoting or suppressing the pathogenesis of certain allergic diseases. Therefore, as they are closely involved in the modulation of IgE-mediated anaphylactic degranulation, CD300 molecules, not only CD300a, but also CD300c and CD300f, and their ligands, should be considered as novel therapeutic targets for anti-allergic therapy, providing a valuable tool for the treatment of allergy and mast cell-associated disorders. Apart from their use as therapeutic targets, their altered expression in allergic patients could help in the understanding of the etiopathogenesis of different allergic diseases, highlighting the importance of further research in this field with the main objective of improving the current therapies.

## Figures and Tables

**Table 1:** Summary: CD300 in mast cells, eosinophils and basophils.

	MAST CELLS	EOSINOPHILS	BASOPHILS	Refs.
**CD300a** **(also named as CMRF-35H, IRp60, IRC1 or IRC2 in humans and CLM-8, LMIR-1 or MAIR-I in mice)**	✓ Constitutively expressed ✓ Eosinophil-derived MBP and EDN down-regulate CD300a expression on CBMCs ✓ Inhibit IgE/FcεRI dependent activation ✓ Self-regulation mechanism by *cis*-interaction of CD300a with PS	✓ Constitutively expressed ✓ Hypoxia and GM-CSF increase CD300a expression ✓ Inhibit Src kinase-dependent activation pathways	✓ Constitutively expressed ✓ Up-regulated in response to IgE/FcεRI cross-linking and stimulation with fMLP, PMA and PMA + ionomycin ✓ Inhibit IgE/FcεRI dependent activation	[57,62,63,64,65,68,70]
**CD300b** **(also named as CD300lb or IREM-3 in humans and CLM-7, LMIR-5, CD300b or mIREM3 in mice)**	✓ Constitutively expressed ✓ Activating role	No available data	No available data	[80]
**CD300c** **(also named as CMRF-35A in humans and CLM-6 in mice)**	✓ Constitutively expressed. ✓ Activating role	No available data	✓ Constitutively expressed ✓ Up-regulated in response to IL-3 ✓ Co-stimulate IgE/FcεRI dependent activation	[42,58]
**CD300d** **(also named as CD300ld in humans and CLM-5, LMIR-4 or MAIR-IV in mice)**	✓ Expressed on mice ✓ LPS-mediated stimulation down-regulate CD300d expression ✓ Activating role	No available data	No available data	[74]
**CD300f** **(also named as CD300lf, IREM-1 or IgSF13 in humans and CLM-1, DIgR2, LMIR-3 or MAIR-V in mice)**	✓ Constitutively expressed ✓ Allergen challenge increase CD300f expression ✓ Inhibitory and activating roles have been demonstrated	✓ Constitutively expressed ✓ Allergen challenge increase CD300f expression ✓ Negative regulator of eotaxin-induced responses ✓ Activating role in innate immune activities of eosinophils	✓ Constitutively expressed	[58,59,74,75,76,77]
**CD300h** **(also named as CLM-3 or LMIR-7 in mice)**	✓ Expressed on mice ✓ Activating role	No available data	No available data	[83]

## Abbreviations

AAAAI	American Academy of Allergy, Asthma and Immunology
Ab	antibody
AD	atopic dermatitis
BMMCs	bone marrow-derived mast cells
CBMCs	cord blood-derived mast cells
DAP	DNAX-activating protein
EDN	eosinophil-derived neurotoxin
ERK	extracellular signal-regulated kinase
FcR	Fc receptor
GM-CSF	granulocyte macrophage-colony stimulating factor
Grb2	growth factor receptor-bound protein 2
HIF	hypoxia inducible factor
Ig	immunoglobulin
IL	interleukin
ITAM	immunoreceptor tyrosine-based activating motif
ITIM	immunoreceptor tyrosine-based inhibitory motif
LPS	lipopolysaccharide
LT	leukotrienes
mAb	monoclonal antibody
MBP	major basic protein
NGF	nerve growth factor
OVA	ovalbumin
PBdMC	peripheral blood-derived mast cells
PCA	passive cutaneous anaphylaxis
PE	phosphatidylethanolamine
PI3K	phosphatidylinositol 3-kinase
PMA	phorbol 12-myristate 13-acetate
PS	phosphatidylserine
SCF	stem cell factor
TGF-β1	transforming growth factor beta-1
Th2	T helper type 2
TNF	tumor necrosis factor
TSLP	thymic stromal lymphopoietin
VEGFA	vascular endothelial growth factor
WT	wild type
